# Case of Injury of the Brain, without a Fracture, Relieved by Application of the Trephine

**Published:** 1792

**Authors:** John Andrews

**Affiliations:** Surgeon in London.


### III. *Case of Injury of the Brain, without a Fracture, relieved by Application of the Trephine. By Mr.* John Andrews, *Surgeon in London.*

ON the 23d of July, 1791, Elizabeth Bell, of Great White Lion Street, thirty-five years of age, rather corpulent, and of a sanguineous temperament, fell down eight or ten stairs, and struck her head upon a boarded floor.

She remained without any medical assistance until the third day after the accident, when I was desired to see her. The bystanders informed me that she had become sick and speechless immediately after the fall, and, after a short interval, comatose. She now discharged her urine involuntarily, and had had a shivering fit a few hours before I saw her: twelve ounces of blood were, therefore, taken from the arm, and a solution of sal. cath. amar. was directed to be given till the bowels were emptied; her whole head was also ordered to be shaven, and a blister was applied to the posterior part of the neck.

On the day following, as her former symptoms were not at all relieved, I thought it advisable to open the temporal artery, which was done done, and with ſome advantage; for her pulſe, which before was very ſlow and oppreſſed, beat a little quicker before I left her, and upon being rouſed, and aſked how ſhe did, ſhe replied, "Better than before." As the cathartic medicine had produced no effect, a purging enema was adminiſtered in the afternoon, and ordered to be repeated, if neceſſary.

The whole head was now more attentively examined both by myſelf and my friend Mr. Blair, who was preſent; but no injury either of the ſcalp or cranium could be diſcovered, though we preſſed pretty forcibly on every part of the head.

On the ſubſequent morning (28th) I was informed ſhe had been attacked in the night with convulſive fits, two of which occurred while I was with her. I obſerved that only the left ſide of her body was affected, her mouth and face on that ſide being diſtorted as in epilepſy; that her head inclined to the left ſide; and that the leg and arm on the ſame ſide (particularly the latter) were contracted and violently agitated.

During this day ſhe had frequent returns of the convulſions, each fit laſting only a few minutes: I therefore drew ten ounces more blood from the arm, and reſolved, if this did not relieve lieve her, to divide the ſcalp on the following day, and ſearch more minutely for a fracture. Upon preſſing the ſcalp with my finger, I obſerved at this time that a little depreſſion was left; but, as this was not a circumſcribed œdema, it could not be regarded as a ſufficient reaſon for making an inciſion, or applying the trephine on any particular part.

The œdema was more univerſal on the ſubſequent morning (the 29th): the convulſions were become frequent and violent; her pulſe was ſlow and more depreſſed; her fæces and urine were ſtill diſcharged involuntarily, and her extremities were becoming very cold. Theſe alarming ſymptoms ſeemed to juſtify a mode of treatment that would otherwiſe not have been adopted: I therefore made an inciſion three or four inches in length on the left parietal and temporal bones; but as no injury was perceived on the denuded bones, the lips of the wound were placed in contact, and kept together by means of ſticking plaſter, in order to accelerate their union. A free inciſion, ſimilar to the former, was then made on the oppoſite ſide, and the bone laid bare, but no preternatural appearance was obſervable, though, on preſſure upon the anterior and inferior edge of the parietal bone, the the patient feemed to fuffer pain, as I judged from her drawing up the right angle of the mouth. Upon this part of the cranium, and upon the edge of the fquamous future, I applied the trephine. As foon as the circular piece of bone was removed, a quantity of extravafated blood (which I fuppofed from its colour to be venous) was feen through the dura mater, diftending that membrane: a fmall oblique puncture was, therefore, made with a lancet, and a table-fpoonful of blood was difcharged by a ftream. A fmall piece of lint was then applied lightly on the dura mater, and the wound dreffed in the ufual way. After the operation her pulfe, which before had been very flow and oppreffed, quickened, and in a few hours beat 120 in a minute. She was ordered to take an opening mixture occafionally, and ftrict attention was paid to her diet.

On the 30th of July, the day after the operation, fhe had feveral more fits, but they were lefs violent and recurred lefs frequently than before; the fame quicknefs of pulfe continued, and fhe flept better than ufual.

On the 31ft, on account of the fulnefs of her pulfe, eight ounces of blood were taken from the arm: ſhe had this day only two fits, and was much relieved from her other complaints.

She continued to get better, nothing very particular happening, until the 4th of Auguſt, when, upon removing the dreſſings, a remarkable elaſtic tumour was diſcovered on the left ſide of the head, extending about three inches in length, and two or more inches in breadth. What could be the cauſe of this ſwelling? If it had been occaſioned by an effuſion of blood under the ſcalp, would it not have appeared much earlier?—Mr. Pott uſed to regard ſuch a puffy riſing of the integuments as a pretty ſure criterion of a fluid under the cranium, immediately beneath the ſwelling. But whatever might be the cauſe of this appearance, it did not remain long; for in little more than a week it gradually diſappeared. The granulations from the dura mater were now uniting with thoſe from the ſcalp, and by the 6th of October the wounds were perfectly healed.

The laſt time I ſaw her (December 10th) ſhe was as entirely free from complaint as ſhe ever had been at any time previous to the accident.

*Great Ruſſell Street, Bedford Square,*

Feb. 22, 1792.

